# Epigenetic control of an oncogenic microRNA, miR-155, by BRCA1

**DOI:** 10.18632/oncotarget.433

**Published:** 2012-01-25

**Authors:** Suhwan Chang, Shyam K. Sharan

**Affiliations:** Mouse Cancer Genetics Program, Center for Cancer Research, National Cancer Institute at Frederick, Frederick, Maryland 21702, USA

Since its identification in 1994, *BRCA1* (*Breast Cancer 1*) has been established as a familial early onset breast and ovarian cancer susceptibility gene [1]. The protein encoded by this gene is involved in diverse cellular processes such as DNA damage repair, cell cycle control, ubiquitination and transcriptional regulation of target genes [2]. Recently, we uncovered a new function of BRCA1, adding another mechanism of tumor suppression by this multifunctional protein [3].

Using a mouse embryonic stem (mES) cell based assay [4], we examined the functional consequences of a BRCA1 variant, R1699Q, found in some cancer patients. One of the functional readouts of this assay is the ability of BRCA1 variants to rescue the lethality of *Brca1 null* mES cells. We found R1699Q to result in a 10-fold reduction in survival these ES cells compared to WT human BRCA1. Interestingly, we found R1699Q to have no effect on DNA damage repair or cell cycle regulation, which are regarded as major functions of BRCA1. Instead, microRNA expression analysis in embryoid bodies generated from WT and R1699Q expressing ES cells, showed that some microRNAs were differentially regulated in R1699Q mutant cells. Discovered in 1993, microRNAs are short cellular RNAs (22nt in average) present in eukaryotic cells and are regarded as important regulators of diverse cellular functions [5]. Generally, microRNAs interact with 3'UTR of target mRNAs by complementary sequence matching and consequently, repress the translation or induce the degradation of target mRNA.

Among the candidate microRNAs identified, we focused on miR-155 because it showed the highest and most consistent increase in the R1699Q mutant cells. miR-155 is a well-known oncogenic microRNA that is encoded by a non-coding RNA BIC (B-cell Insertion Cluster). A viral insertion on this gene was shown to induce lymphoma. In addition, B-cell specific expression of miR-155 in mouse caused B-cell lymphoma [6]. Based on these findings, we hypothesized that the possible repression of an oncogenic microRNA by BRCA1 may be a novel mode of tumor suppression. Indeed, a series of experiments supported this idea and we found that BRCA1 recruits HDAC2 complex to the miR-155 promoter. Consequently, the promoter is epigenetically silenced through the deacetylation of H2A and H3 histones. More importantly, our study also showed the up-regulation of miR-155 in BRCA1 deficient or BRCA1 mutant human tumors. Finally, based on our observation that the knockdown of miR-155 in a Brca1 mutant tumor cell line attenuates *in vivo* tumor growth, we proposed a therapeutic potential of anti-miR-155 agent for BRCA1-associated breast cancer.

Even though we have described the mechanism of BRCA1-mediated silencing of miR-155 promoter in detail, there are several issues that need to be resolved to fully understand the complex mechanism of the BRCA1 mediated microRNA regulation and its effect on the breast cancer. First, what is the role of miR-155 in BRCA1-meidated tumorigenesis? Because a single microRNA is predicted to regulate up to 200 mRNAs [7], it is important to identify the targets of miR-155 in breast epithelial cells that contribute to tumorigenesis. Second, what other microRNAs are epigenetically regulated by BRCA1? Identification of these other microRNAs and their targets will be a key step towards understanding the BRCA1 regulated microRNA network and its role in tumorigenesis.

Interestingly, we found that the knockdown of BRCA1 results in a 2 to 3-fold increase in miR-155 levels. In contrast, we observed 50 to 150-fold increase in miR-155 in human breast cancer cell lines or tumor samples suggesting that this increase may not be caused only by BRCA1 loss. We hypothesize that other transcription factors may activate the miR-155 promoter after it is epigenetically activated due to the loss of BRCA1. Identification of such regulatory factors will help us to fully understand the regulations of this oncogenic microRNA during the tumorigenesis. One of the possible regulatory factors is TGFβ, which has been shown to induce miR-155 expression via SMAD4 [8]. Finally, as a cautionary note, it is worth mentioning that anti-miR-155 agents need to be carefully tested *in vivo* for their therapeutic effects. Functional studies using miR-155 knockout mice have shown that miR-155 is essential for normal immune function [9]. Considering the systemic inhibition of miR-155 will abrogate the immune function that can be advantageous for the tumor growth, anti-miR-155 agents may have to be delivered directly to the tumor cells.

In summary, while our finding that BRCA1 epigenetically silences an oncogenic microRNA is quite significant, many questions remain to be answered. We hope that our efforts to answer these questions will lead to a better understanding of the tumor suppressor function of BRCA1 and allow us to develop novel therapeutic strategies for the breast cancer.
